# Lymph Nodal Infarction Simulating Acute Appendicitis

**Published:** 2011-07-30

**Authors:** Maham Zaman, Samina Zaman, Lubna Ijaz, Mahwish Hussain, Ghazala Hanif, Bilal Mirza, Afzal Sheikh

**Affiliations:** Department of Pediatric Surgery, The Children's Hospital and the Institute of Child Health Lahore, Pakistan; Department of Pediatric Surgery, The Children's Hospital and the Institute of Child Health Lahore, Pakistan; Department of Pediatric Surgery, The Children's Hospital and the Institute of Child Health Lahore, Pakistan; Department of Pediatric Surgery, The Children's Hospital and the Institute of Child Health Lahore, Pakistan; 1 Department of Histopathology, The Children's Hospital and the Institute of Child Health Lahore, Pakistan

**Keywords:** Lymph node infarction, Acute appendicitis, Pain right iliac region

## Abstract

A number of diseases can present as acute right iliac region pain. Lymph node infarction, located adjacent to the cecum, mimicking acute appendicitis in a 13-year-old boy is presented here.

## INTRODUCTION

A number of diseases have been reported to present with acute pain in right iliac region, simulating acute appendicitis [1]. Infarction of lymph nodes has been reported with many neoplastic and non-neoplastic conditions. In patients with malignant lymphoma, infarction of the lymph nodes has been reported quite frequently [2, 3]. Lymph node infarction located adjacent to the cecum is a condition reported hitherto, the purpose of which is to add another cause of pain abdomen simulating acute appendicitis.

## CASE REPORT

A 13-year-old boy presented with acute pain in right iliac region for a day. The pain initially felt in the periumbilical region. There was no associated fever, anorexia, nausea or vomiting. On palpation of the abdomen, there was muscle guarding, localized abdominal tenderness in the right iliac fossa with rebound tenderness. Laboratory tests showed hemoglobin 11gm/dl and WBC count 13700/cmm with predominant polymorphonuclear cells. Ultrasound of abdomen was reported as normal. The score on Alvarado scale was 7, indicating probable appendicitis.

At operation, normal looking appendix was observed. Appendectomy was done. The Meckel’s diverticulum was not present. Further exploration revealed an enlarged lymph node adjacent to the cecal wall. The connective tissue over the lymph node was incised that revealed a necrosed lymph node (Fig. 1). Specimens of the appendix and lymph node were sent for histopathology. The histopathology report showed an appendix measuring 6x0.5cm with lymphoid hyperplasia but no signs of acute inflammation. The lymph node biopsy revealed hemorrhagic necrosis of its parenchyma. The few preserved areas of lymph node were free of any malignant cells (Fig. 2, 3). The patient had an uneventful recovery. He is counseled for diligent follow-up with repeated ultrasound abdomen. He is doing well at 6 months follow-up. 

**Figure:**
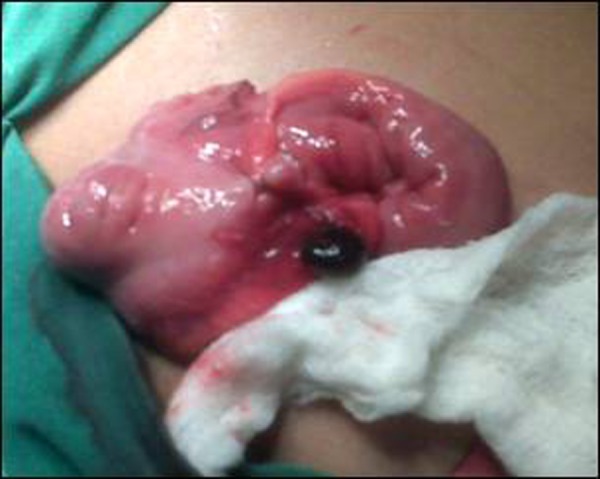
Figure 1: An infarcted lymph node just adjacent to cecum.

**Figure:**
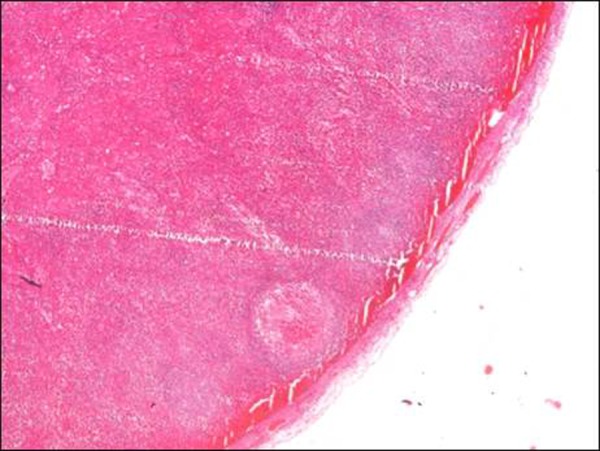
Figure 2: Showing a thin rim of viable subcapsular lymphoid tissue with hemorrhage in the marginal sinus (x40)

**Figure:**
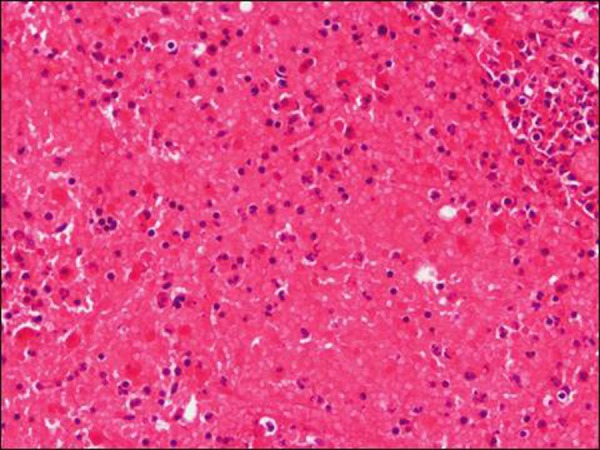
Figure 3: Showing extensive necrosis of medullary and cortical lymphoid cells with extravasated red blood cells (x 400).

## DISCUSSION

Pain in RIF has many differential diagnoses [1]. The Alvarado score in our case was 7 which had a high probability of acute appendicitis, however, a distinct infarcted lymph node lying adjacent to the cecal wall was the only pathology observed in presence of histologically normal appendix. On the other hand, the histopathology of the infarcted lymph node showed extensive hemorrhagic necrosis.

There are multiple causes of lymph node infarction and can be categorized as iatrogenic, non-neoplastic and neoplastic. Lymphoma is a frequently reported neoplastic cause of lymph node infarction with an incidence of 32-89%.The lymphoma may be synchronously present or develop after months to years of initial event. Therefore a long term follow up is advised in such cases [2, 3].

To the best of our knowledge, such a presentation of infarcted lymph node is not reported before. In addition infarction of lymph node must be kept in differential diagnosis of acute right iliac region pain. 

## Footnotes

**Source of Support:** Nil

**Conflict of Interest:** None declared
